# Diagnosis, Treatment Patterns and Eradication Success for *Helicobacter pylori* Infections in China: A Retrospective Observational Real‐World Study

**DOI:** 10.1002/jgh3.70232

**Published:** 2025-10-10

**Authors:** Changqin Xu, Wensheng Pan, Yan Zhao, Pengfei Li, Fang Zhou, Li Xie, Hongwei Xu

**Affiliations:** ^1^ Department of Gastroenterology Shandong Provincial Hospital Affiliated to Shandong First Medical University Jinan China; ^2^ Department of Gastroenterology Zhejiang Provincial People's Hospital Hangzhou China; ^3^ Department of Gastroenterology Shanghai Tenth People's Hospital Shanghai China; ^4^ TDC Takeda APAC Biopharmaceutical R&D Ltd Shanghai China; ^5^ China Medical Team, Takeda (China) International Trading Co. Ltd. Shanghai China

**Keywords:** bismuth quadruple therapy, China, *Helicobacter pylori*, real world

## Abstract

**Background and Aim:**

In Mainland China, *Helicobacter pylori* infection is prevalent in about 50% of adults. However, there is limited real‐world evidence on its diagnosis and treatment patterns. This study investigated diagnosis, treatment patterns, and effectiveness of eradication treatment in patients with *Helicobacter pylori* infections in China.

**Methods and Results:**

This retrospective, observational, real‐world study used data from electronic medical records databases from three hospitals in Shanghai City, Shandong Province, and Zhejiang Province. Patients (≥ 18 years) with an *H. pylori* infection diagnosis between January 01, 2019, and December 31, 2021, were included. Of the 22 971 included patients (mean [standard deviation] age: 44.2 [14.2] years; female: 54.6%), 13 233 (57.6%) received eradication therapy and 1626 (7.1%) received efficacy evaluation testing. Overall, 85.4% of patients received bismuth‐based quadruple therapies (BQTs), and 7.6% and 7.1% received triple and dual therapies, respectively. For BQT, the most common antibiotic combinations included amoxicillin and clarithromycin (38.7%), amoxicillin and furazolidone (18.4%), and amoxicillin and levofloxacin (17.8%). A 14‐day treatment duration was most common for BQT; however, from 2020 to 2021, the number of patients with a 14‐day treatment duration decreased. BQT had the highest eradication rate (76.7%), followed by dual (57.0%) and triple (48.4%) therapies. Eradication rates for amoxicillin and clarithromycin and for amoxicillin and furazolidone BQTs were 76.1% and 87.4%, respectively.

**Conclusions:**

Treatment of *H. pylori* infections in China is fairly consistent with the national guidelines, with room for further improvement and standardization, especially with respect to antibiotic combinations, treatment duration, and efficacy evaluation testing.

**Trial Registration:** ClinicalTrials.gov identifier: NCT05073367

## 1 Introduction

The World Health Organization's International Agency for Research on Cancer classifies *Helicobacter pylori* ( *H. pylori* ), a gastric bacterial pathogen, as a human carcinogen [1]. *H. pylori* infection is linked to almost 90% of distal gastric cancers globally [2]. *H. pylori* is also the most common cause of chronic gastritis and peptic ulcers [3] and is associated with other conditions such as colon cancer, idiopathic thrombocytopenic purpura, iron deficiency anemia, and vitamin B12 deficiency [4, 5, 6]. While numbers vary across countries, more than half of the global population is infected by this pervasive pathogen [7]. In Mainland China, it is estimated to be prevalent in almost half of the adult population [8, 9]. Although the *H. pylori* infection rate shows a decreasing trend in China [9], many individuals remain undiagnosed due to being asymptomatic and/or because of the limited awareness of pathogen screening [10].

Eradication of *H. pylori* can reduce the risk of gastric cancer [11, 12]. Globally, *H. pylori* infection treatment guidelines recommend a “test‐and‐treat” strategy, which comprises noninvasive testing for *H. pylori* in patients with dyspeptic symptoms and eradication of the infection whenever detected [13]. Urea breath test (UBT) is the most recommended noninvasive diagnostic test to detect *H. pylori* [14]. However, findings from surveyed physicians in China indicate that up to 40% do not follow guideline recommendations for diagnosis [15].

Currently, bismuth‐based quadruple therapy (BQT) comprising a proton pump inhibitor (PPI), bismuth, and two antibiotics, is the recommended first‐line therapy for *H. pylori* eradication in most countries, although high‐dose dual therapy (PPI + antibiotic) is also gaining attention [13]. Additionally, vonoprazan, a novel potassium‐competitive acid blocker (PCAB), has demonstrated superior inhibition of gastric acid secretion without the need for acid activation compared with PPIs [16]. In China, the recommended first‐ and second‐line therapies for *H. pylori* eradication are BQT or high‐dose dual therapy [17]. Since 2012, standard triple therapy (PPI + two antibiotics) is no longer recommended in China as first‐ or second‐line therapy due to increased antibiotic resistance [18].

While several randomized clinical trials (RCTs) have been conducted in China to determine the comparative efficacies of different eradication regimens, real‐world evidence investigating the diagnosis and treatment patterns of *H. pylori* infections remains limited. A real‐world study on the status of *H. pylori* infections in China used electronic medical record (EMR) data from a provincial medical institution in Zhejiang Province between June 2018 and May 2019 [19]. However, it only focused on standard 14‐day quadruple therapy and did not consider standard triple therapy and levofloxacin‐containing therapy, which may still be prevalent in real‐world settings.

Up‐to‐date and comprehensive understanding of *H. pylori* diagnosis and its treatment patterns as well as the eradication rates of treatments prescribed can support evidence‐based treatment strategies for *H. pylori* eradication, with the overall goal of improving patient outcomes. This retrospective observational real‐world study was therefore conducted to investigate diagnosis and treatment patterns as well as the effectiveness of eradication treatment in patients with *H. pylori* infections in China.

## 2 Materials and Methods

### 2.1 Study Design and Patient Population

This multicenter, retrospective study (ClinicalTrials.gov NCT05073367) used de‐identified patient‐level data from pre‐existing EMR databases from three hospitals in Shanghai City, Shandong Province, and Zhejiang Province in China.

**FIGURE 1:**
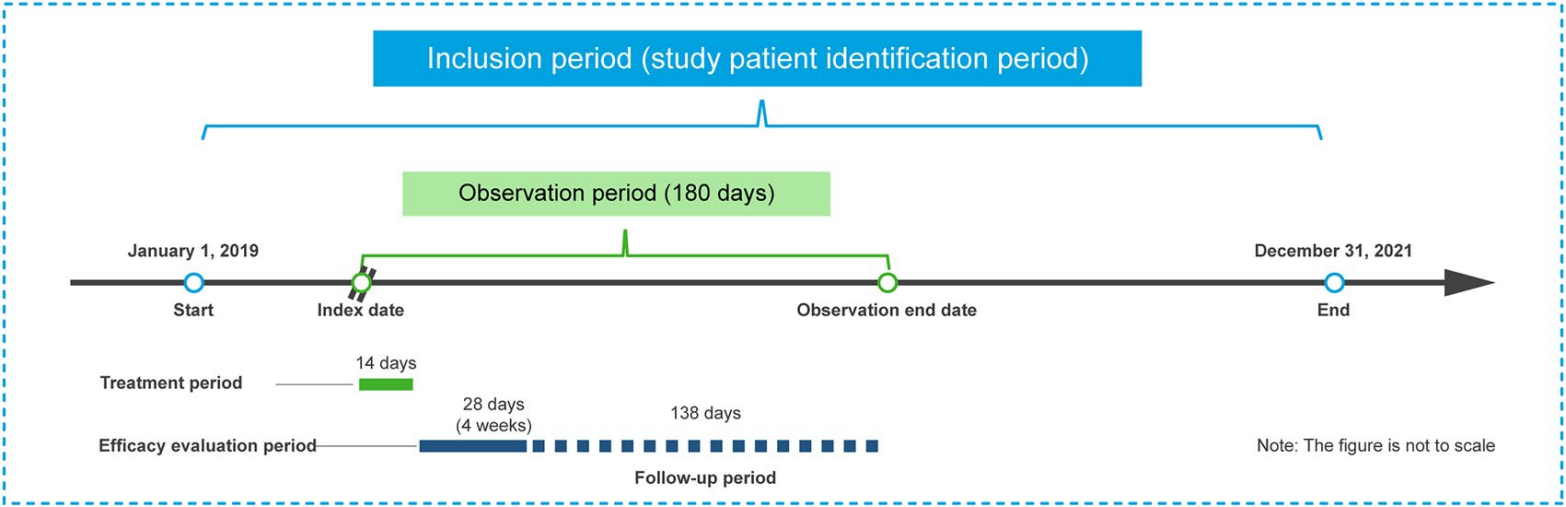
Study design.

Patients aged ≥ 18 years at the index date, with a record of *H. pylori* infection diagnosis or a positive result in *H. pylori* diagnostic testing between January 01, 2019, and December 31, 2021, were included. The index date was defined as the first diagnosis date or the first date of a positive result in *H. pylori* diagnostic testing (whichever was first during the patient identification period). The postindex observation period was a maximum of 180 days (including the 10–14‐day treatment period, 28‐day efficacy evaluation period [14], and 138‐day follow‐up period) (Figure 1). All patients were sampled using random sampling. Ethics statements for the study are provided in the Supporting Information.

#### 2.1.1 Analysis Sets

Four analysis sets were defined. The all‐patients enrolled (ENR) set included all enrolled patients who satisfied the eligibility criteria. The diagnostic analysis set (DAS) included all enrolled patients who satisfied the study eligibility criteria and had a diagnostic testing record. The treatment analysis set (TAS) included all enrolled patients who satisfied the study eligibility criteria and had a record of eradication therapy, and the efficacy evaluation analysis set (EEAS) consisted of patients in the TAS who had a record of efficacy evaluation testing.

### 2.2 Key Endpoints

The primary endpoint (analyzed using the TAS) included the proportion of each eradication regimen initially prescribed after the index date.

Key secondary endpoints included the proportions of the following: duration of each eradication therapy from the date of prescription until the termination of eradication therapy (TAS); each diagnostic test used before the prescription of eradication therapy (DAS); each efficacy evaluation testing after the termination of eradication therapy (EEAS); and duration (≤ 4 weeks or > 4 weeks) from the termination of eradication therapy until efficacy evaluation testing (EEAS). The exploratory endpoint was the eradication rate of *H. pylori* in positive patients receiving standard treatment (EEAS).

For all endpoints, subgroup analyses were performed by the year that the treatment was initially prescribed (2019, 2020, or 2021).

#### 2.2.1 Variables

Eradication regimens were identified according to the combination of drugs that were prescribed on the same day. Treatment duration was categorized as 7, 10, or 14 days (the drugs were prescribed for), or others. *H. pylori* diagnostic testing included noninvasive testing (UBT, fecal antigen testing, and serological testing) and invasive testing (rapid urease test [RUT], histology, polymerase chain reaction, and culture) in accordance with national recommendations in use at the time of the study [14].

Efficacy evaluation testing was defined as *H. pylori* testing performed after the 28 days of the efficacy evaluation period after treatment completion; this testing could extend to the follow‐up period until the end of the observation period. The termination of medication until the efficacy evaluation testing was defined as the period between the last day of taking the medication for eradication therapy and the day of receiving efficacy evaluation testing. The eradication rate was equal to the number of patients with a negative result in the efficacy evaluation testing divided by the number of patients who received efficacy evaluation testing.

### 2.3 Statistical Analysis

All analyses were descriptive, with categorical variables reported using frequency and percentage and continuous variables reported as mean ± standard deviation (SD) or median (interquartile range). Missing data for each variable were summarized with frequency and percentage of missing values. Data analyses were performed using R (version 4.2.3) (R Core Team, Vienna, Austria). As this study is descriptive in nature, *p* values have not been reported.

## 3 Results

Of the total of 23,000 patients screened, 22,971 fulfilled the eligibility criteria and were included in the ENR and 15,712 were included in the DAS. A total of 13,233 patients were treated with eradication therapy (TAS); 9738 received no eradication therapy and 1626 were in the EEAS (Table S1). In the ENR, the mean (SD) age of patients was 44.2 (14.2) years; more than half were female (*n* = 12 535; 54.6%) (Table S2). Patients were spread evenly across three participating hospitals (Shanghai City [34.8%], Shandong Province [30.5%], and Zhejiang Province [34.7%]).

### 3.1 Distribution of Eradication Therapies

Most patients received BQT (85.4%), followed by triple therapies (7.6%) and dual therapies (7.1%) (Table 1). Among patients receiving BQT, the most common antibiotic combination was amoxicillin and clarithromycin (38.7%). Most (60.3%) patients were prescribed clarithromycin and levofloxacin as triple therapies, and among dual‐therapy users, levofloxacin (44.9%) and ornidazole (42.6%) were the most frequently prescribed antibiotics (Table 1).

**TABLE 1:** Distribution of eradication therapy (TAS).

	Total (*N* = 13 233)
	*n* (%)^a^
Total count of all standard eradication therapies used	13 234^b^
Bismuth quadruple therapy (bismuth + acid suppressants + antibiotic 1 + antibiotic 2)	11 301 (85.4)
Amoxicillin and clarithromycin–based therapy	4376 (38.7)
Amoxicillin and furazolidone–based therapy	2074 (18.4)
Amoxicillin and levofloxacin–based therapy	2007 (17.8)
Clarithromycin and levofloxacin–based therapy	1337 (11.8)
Levofloxacin and metronidazole–based therapy	400 (3.5)
Clarithromycin and furazolidone–based therapy	372 (3.3)
Levofloxacin and ornidazole–based therapy	128 (1.1)
Clarithromycin and ornidazole–based therapy	127 (1.1)
Other antibiotic–based bismuth quadruple therapy	480 (4.3)
Triple therapy (acid suppressants + antibiotic 1 + antibiotic 2)	999 (7.6)
Clarithromycin and levofloxacin–based therapy	602 (60.3)
Levofloxacin and ornidazole–based therapy	106 (10.6)
Amoxicillin and clarithromycin–based therapy	83 (8.3)
Other antibiotic–based triple therapy	208 (20.8)
Dual therapy (acid suppressants + antibiotic)	934 (7.1)
Levofloxacin‐based therapy	419 (44.9)
Ornidazole‐based therapy	398 (42.6)
Clarithromycin‐based therapy	77 (8.2)
Other antibiotic–based dual therapy	40 (4.3)

Abbreviation: TAS, treatment analysis set.

^a^ The percentage is based on either the total usage count of all standard eradication therapies taken by eligible patients for the overall therapy results or on the total usage count of bismuth quadruple therapy/triple therapy/dual therapy for individual antibiotic combination results.

^b^ One patient changed the treatment pattern from triple therapy (ilaprazole, clarithromycin, levofloxacin) to dual therapy (ilaprazole, ornidazole) after recurrence.

Among BQTs, the most frequently used PPI was pantoprazole (60.3%), followed by rabeprazole (18.4%), omeprazole (10.7%), and esomeprazole (9.5%).

When analyzed by the year when treatment was initially prescribed, the percentage of patients using BQTs decreased from 97.2% in 2019 to 80.0% in 2020 and 78.7% in 2021. The percentage of patients who used triple therapy fluctuated, increasing from 2.3% in 2019 to 13.4% in 2020 but decreasing to 7.4% in 2021. The percentage of patients using dual therapy showed a steady increase from 0.4% in 2019 to 6.7% in 2020 and 13.9% in 2021 (Figure S1).

Analysis of antibiotic combination trends over the 3 years revealed that for BQTs, the use of amoxicillin and clarithromycin combination decreased from 41.2% in 2019 to 40.1% in 2020 and 34.5% in 2021. Similarly, the use of amoxicillin and levofloxacin decreased from 23.4% in 2019 to 12.9% in 2021. In contrast, the use of the amoxicillin and furazolidone combination increased from 15.8% in 2019 and 14.5% in 2020 to 24.9% in 2021 (Table S3). Among triple therapies, there was a sharp decline from 2019 to 2021 in the use of amoxicillin and clarithromycin (41.0% to 6.4%) and amoxicillin and levofloxacin (21.0% to 7.0%), while clarithromycin and levofloxacin–based triple therapies increased from 28.6% in 2019 to 83.9% in 2020 and decreased to 32.1% in 2021. Among dual therapies, an increase over the 3 years was only observed with ornidazole‐based dual therapy, which showed a sharp increase from 5.3% in 2019 to 61.4% in 2021 (Table S3).

### 3.2 Treatment Duration of Eradication Therapy

Among patients who received BQTs, the majority had a treatment duration of 14 days (90.2%). Similarly, among those who received triple therapies, 76.8% had a treatment duration of 14 days. However, most (82.4%) patients on dual therapies had a treatment duration of > 14 days (Table 2).

**TABLE 2:** Treatment duration of eradication therapy (TAS).

	Total (*N* = 13 233)
	*n* (%)
Total count of all standard eradication therapies used (with information on treatment duration)	10 903
Bismuth quadruple therapy	9855 (100.0)
Duration (days)	
< 10	254 (2.6)
= 10	35 (0.4)
> 10 and < 14	249 (2.5)
= 14	8885 (90.2)
> 14	432 (4.4)
Triple therapy	521 (100.0)
Duration (days)	
< 10	18 (3.5)
= 10	4 (0.8)
> 10 and < 14	9 (1.7)
= 14	400 (76.8)
> 14	90 (17.3)
Dual therapy	527 (100.0)
Duration (days)	
< 10	12 (2.3)
> 10 and < 14	5 (1.0)
= 14	76 (14.4)
> 14	434 (82.4)

Abbreviation: TAS, treatment analysis set.

**FIGURE 2:**
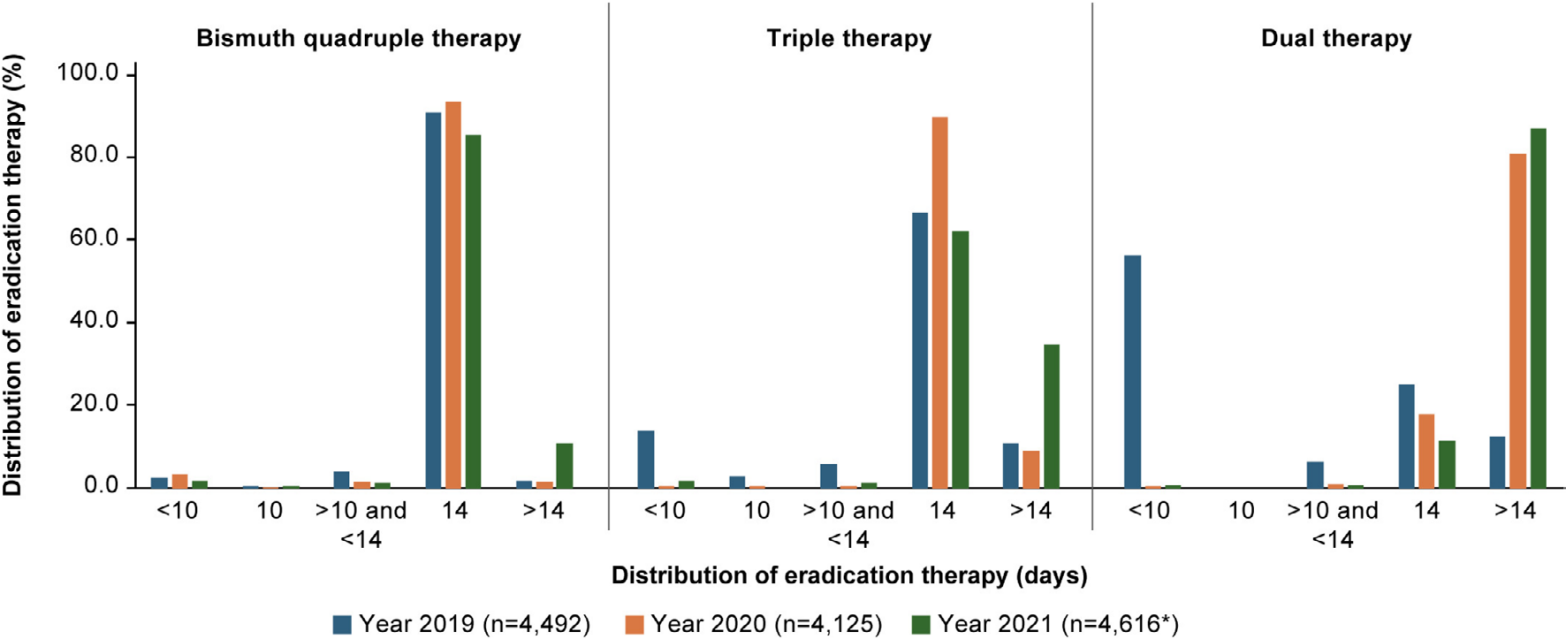
Duration of eradication treatment by the year when treatment was initially prescribed (TAS). TAS, treatment analysis set. *One patient changed the treatment pattern from triple therapy (ilaprazole, clarithromycin, levofloxacin) to dual therapy (ilaprazole, ornidazole) after recurrence.

In the subgroup analysis by year, while a 14‐day treatment duration was most common for BQTs and bismuth triple therapies across the years, there was a decrease in the number of patients with a 14‐day treatment duration from 2020 to 2021, accompanied by an increase in the > 14‐day treatment duration over the same period for both therapies (Figure 2). For dual therapies, in 2019, a treatment duration of < 10 days was most common (56.3%); however, in 2020 and 2021, a > 14‐day treatment duration was observed in most patients (80.8% and 87.2%, respectively).

### 3.3 Diagnostic Testing Used Before Eradication Therapy

Among patients with a record of diagnostic testing, almost all used noninvasive diagnostic testing (i.e., UBT), including 13C‐UBT (76.8%) and, to a lesser extent, 14C‐UBT (23.2%) (Table 3). The percentage of patients using 13C‐UBT increased from 47.5% in 2019 to 73.2% in 2020 and 100.0% in 2021. Meanwhile, the percentage of patients using 14C‐UBT decreased from 52.5% in 2019 to 26.8% in 2020 and 0% in 2021 (Figure S2).

**TABLE 3:** Diagnostic testing used before eradication therapy (DAS) and efficacy evaluation testing used after eradication therapy (EEAS).

	*n* (%)
Before eradication therapy	
The total count of diagnostic testing	15 713^a^
Invasive testing	
RUT	1 (0.01)
Noninvasive testing	
UBT	15 712 (100.0)
13C‐UBT	12 068 (76.8)
14C‐UBT	3644 (23.2)
After eradication therapy	
The total count of efficacy evaluation testing	1426
Noninvasive testing	
UBT	1426 (100.0)
13C‐UBT	1031 (72.3)
14C‐UBT	395 (27.7)

Abbreviations: DAS, diagnostic analysis set; EEAS, efficacy evaluation analysis set; RUT, rapid urease test; UBT, urea breath test.

^a^ A patient may have one or more diagnostic tests. One patient had both RUT and UBT data at the index date.

### 3.4 Efficacy Evaluation Testing Used After Eradication Therapy

All patients with an efficacy evaluation testing performed > 4 weeks after termination of eradication treatment used UBT (Table 3). Most patients used 13C‐UBT (72.3%). The percentage of patients using 13C‐UBT increased from 53.7% in 2019 to 69.9% in 2020 and 100.0% in 2021 (Figure S3). Conversely, the use of 14C‐UBT decreased from 46.3% in 2019 to 30.1% in 2020 and 0% in 2021.

### 3.5 Duration From the Termination of Medication Until the Efficacy Evaluation Testing

For all patients with a record of eradication therapy and with an efficacy evaluation testing (*n* = 1626), the mean (SD) duration from medication termination until efficacy evaluation testing was 7.00 (4.37) weeks (Table 4). Approximately 12.3% of patients had a duration of ≤ 4 weeks between medication termination and efficacy evaluation testing, whereas 87.7% had a duration of > 4 weeks.

**TABLE 4:** Duration from medication termination until efficacy evaluation testing (EEAS).

	Total
The total count of efficacy evaluation testing (*n*)	1626
Duration from the termination of medication until the efficacy evaluation testing (weeks)	
Mean (SD)	7.00 (4.4)
Median	5.4
Q1, Q3	4.7, 7.9
Min, max	0.1, 25.0
Total count of efficacy evaluation testing by duration categories	*n* (%)^a^
≤ 4 weeks	200 (12.3)
> 4 weeks	1426 (87.7)
> 4 and ≤ 8 weeks	1032 (63.5)
> 8 and ≤ 12 weeks	212 (13.0)
> 12 weeks	182 (11.2)

Abbreviations: EEAS, efficacy evaluation analysis set; max, maximum; min, minimum; Q, quartile; SD, standard deviation.

^a^ Percentage calculated as the count of duration divided by the total count of efficacy evaluation testing.

When stratified by the year of initial treatment prescription, the duration from medication termination until efficacy evaluation testing was ≥ 4 weeks in most patients (Figure S4) and fairly stable throughout (mean [SD]: 7.0 [4.6], 7.0 [4.2], and 7.1 [4.3] weeks in 2019, 2020, and 2021, respectively). The percentage of patients with a ≤ 4‐week duration from medication termination to efficacy evaluation testing reduced from 16.6% in 2019 to 9.3% in 2021.

### 3.6 *H. pylori* Eradication Rate

Among patients with a record of eradication therapy and with an efficacy evaluation testing performed > 4 weeks after treatment termination (*n* = 1426), the overall eradication rate was 72.1% (Table 5). The rate of eradication was the highest among patients receiving BQTs (76.7%), followed by dual (57.0%) and triple (48.4%) therapies. Among the most used BQTs, the antibiotic combination with the highest eradication rate was amoxicillin and furazolidone (87.4%). The eradication rates of the most used antibiotic combinations for triple and dual therapies were 45.8% for clarithromycin and levofloxacin–based regimens and 59.6% for levofloxacin‐based therapies, respectively.

**TABLE 5:** Eradication rate of *H. pylori* –positive patients by eradication therapy received (EEAS).

	Total (*N* = 1426)	
	Total	Success
	*n* (%)^a^	*n* (%)^b^
Total usage count of all standard eradication therapies	1426	1028 (72.1)
Bismuth quadruple therapy	1146 (80.4)	879 (76.7)
Amoxicillin and clarithromycin–based therapy	514 (44.9)	391 (76.1)
Amoxicillin and furazolidone–based therapy	207 (18.1)	181 (87.4)
Amoxicillin and levofloxacin–based therapy	204 (17.8)	159 (77.9)
Clarithromycin and levofloxacin–based therapy	104 (9.1)	72 (69.2)
Clarithromycin and furazolidone–based therapy	58 (5.1)	41 (70.7)
Other bismuth quadruple therapy^c^	59 (5.1)	35 (59.3)
Triple therapy	122 (8.6)	59 (48.4)
Clarithromycin and levofloxacin–based therapy	107 (87.7)	49 (45.8)
Amoxicillin and clarithromycin–based therapy	9 (7.4)	4 (44.4)
Other triple therapy^d^	6 (4.9)	6 (100.0)
Dual therapy	158 (11.1)	90 (57.0)
Levofloxacin‐based therapy	109 (69.0)	65 (59.6)
Ornidazole‐based therapy	27 (17.1)	13 (48.1)
Other dual therapy^e^	22 (13.9)	12 (54.5)

Abbreviation: EEAS, efficacy evaluation analysis set.

^a^ Percentage of therapy based on total usage count of all standard eradication therapies taken by eligible patients or on the total usage count of bismuth quadruple/triple/dual therapy for individual antibiotic combination results.

^b^ Success percentage calculated by dividing the number of patients with a positive result for *H. pylori* eradication by the number of patients receiving any eradication therapy or specific (bismuth quadruple/triple/dual) therapy.

^c^ Other antibiotic combinations of bismuth quadruple therapies include levofloxacin and metronidazole (eradication rate: 76.0% [19/25]), clarithromycin and metronidazole (66.7% [6/9]), clarithromycin and ornidazole (0% [0/8]), clarithromycin and minocycline (71.4% [5/7]), amoxicillin and metronidazole (25.0% [1/4]), amoxicillin and ornidazole (100% [1/1]), and minocycline and ornidazole (100% [1/1]).

^d^ Other antibiotic combinations of triple therapies include amoxicillin and metronidazole (eradication rate: 100% [2/2]), clarithromycin and minocycline (100% [1/2]), minocycline and ornidazole (100% [1/2]), amoxicillin and levofloxacin (100% [1/2]), and levofloxacin and metronidazole (100% [1/2]).

^e^ Other antibiotic‐containing dual therapies include clarithromycin (eradication rate: 43.7% [7/16]), amoxicillin (80% [4/5]), and furazolidone (100% [1/1]).

Overall, the eradication rate of all standard eradication therapies was 77.9% in 2019, which declined to 61.0% in 2020 and increased to 75.0% in 2021. In 2019, triple therapy and BQT had eradication rates of 78.6% and 77.9%, respectively. In 2020, these decreased to 46.3% and 67.6%, respectively, and the usage of dual therapies gained traction, with an eradication rate of 50.0%. In 2021, BQTs had the highest eradication rate at 82.8%, followed by dual therapies at 61.2% and triple therapies at 39.3% (Figure S5).

## 4 Discussion

In this real‐world study of patients with *H. pylori* infections in China, we analyzed diagnosis and treatment patterns and the effectiveness of eradication treatment from 2019 to 2021. We found that most patients (approximately 85%) with *H. pylori* infections received BQT, with a standard 14‐day treatment duration in 90% of these patients. Triple and dual therapies were less common and observed in less than 10% of patients. While most patients on BQT used the antibiotic combination of amoxicillin and clarithromycin, followed by amoxicillin and furazolidone, the eradication rate was higher for the combination of amoxicillin and furazolidone (87%), followed by amoxicillin and levofloxacin (78%) and amoxicillin and clarithromycin (76%). Diagnostic testing and efficacy evaluation testing for *H. pylori* infections were most commonly conducted using 13C‐UBT, and for most patients, efficacy evaluation testing was done > 4 weeks after medication termination. The overall *H. pylori* eradication rate across therapies and indications was 72%. BQT had the highest eradication rate (77%), followed by dual (57%) and triple (48%) therapies.

We compared our findings with recommendations in the 5th Chinese National Consensus Report on the management of *H. pylori* [14], which were the treatment guidelines in use at the time of the study.

The observed predominance of BQTs is consistent with its recommendations as the main empirical therapy for patients with *H. pylori* infections. A small proportion of patients used high‐dose dual therapies, although an upward trend was observed from 2019 to 2021. This is likely because high‐dose dual therapies were included only in the updated 2022 guidelines [17]. Triple therapy was also used to some extent; however, current guidelines strongly recommend BQTs over triple therapy [17]. Notably, the use of triple therapy decreased by almost half from 2020 to 2021.

Amoxicillin and clarithromycin was the most prevalent antibiotic combination in BQTs, which aligned with the treatment recommendations. However, the use of amoxicillin and clarithromycin–based quadruple therapies showed a declining trend over the study period, reducing from 41% in 2019 to 35% in 2021, while the use of amoxicillin and furazolidone–based therapies increased from 16% to 25% of patients in 2021. This may reflect increasing resistance to clarithromycin in China, where furazolidone is recommended only for treatment‐resistant *H. pylori* infection owing to the risk of side effects [17]. Some discrepancies with guidelines were observed, as five of the eight most prescribed antibiotic combinations for BQT (clarithromycin and levofloxacin, levofloxacin and metronidazole, clarithromycin and furazolidone, levofloxacin and ornidazole, and clarithromycin and ornidazole) are not part of the seven recommended antibiotic combination regimens [14]. In fact, levofloxacin‐containing regimens, which were commonly observed in the current study, are recommended by guidelines for rescue therapy and not as initial treatment [14]. Additionally, ornidazole is typically not recommended as part of standard *H. pylori* eradication therapy due to concerns regarding antibiotic resistance and potential adverse effects, particularly in pediatric patients. While it may be included in alternative sequential or quadruple therapy regimens under specific circumstances, it is not considered a first‐line or preferred agent [20].

Other findings such as the 14‐day treatment duration among most patients receiving BQTs and UBT being the most common testing method for diagnosis and efficacy evaluation were consistent with guidelines [17], with some discrepancies. The slight decline in the prevalence of the 14‐day treatment duration from 2019 to 2021 accompanied by an increase in the prevalence of the > 14‐day treatment duration may reflect a trend of extending treatment duration to improve eradication rates in the presence of high antibiotic resistance. Furthermore, most patients on dual therapy had a treatment duration exceeding the recommended 14 days. We also observed that 12% of patients did not adhere to the recommended 4‐week gap between medication termination and the efficacy evaluation.

Taken together, while our findings suggest that the diagnosis and treatment of *H. pylori* infections in China are fairly consistent with guidelines and appear to have improved over the years, there is room for further improvement and standardization in clinical practice across hospitals in the country, especially with respect to antibiotic combinations, treatment duration, and efficacy evaluation testing.

The study conducted by Song et al. [21] in Shandong Province examined the patterns and trends of antibiotic consumption in China from 2012 to 2017. The findings revealed a substantial decline in overall antibiotic usage during this period. However, the preference for clarithromycin and levofloxacin remained consistent. This observation aligns with that in the current study, where clarithromycin and levofloxacin are frequently advised to eradicate *H. pylori* . Comparisons with the published literature reflect changes in the *H. pylori* treatment landscape globally and in China. A nationwide survey of physicians found that the percentage of BQTs was 16% in 2014 and 57% in 2017 [15]. In the current study, 85% of patients had received BQT, indicating that the percentage of patients being prescribed BQTs has increased over time, consistent with updates in treatment guidelines [14].

The eradication rate observed for BQTs (77%) was comparable with that observed in a real‐world study in Eastern China between 2018 and 2019, which was 76.6% with the 14‐day quadruple therapy [19]. Another study in China has also reported high eradication rates with amoxicillin‐containing quadruple regimens (amoxicillin and clarithromycin [84%], amoxicillin and furazolidone, and amoxicillin and levofloxacin [79% each]) [19]. Similar higher eradication rates have been reported in European and Asian populations [22]. However, of note, furazolidone is prohibited in Europe and the United States due to its carcinogenic effects [23, 24]. Most BQTs in the current study and all in the study by Yan et al. were PPI‐based regimens. PCAB‐based BQT, including vonoprazan‐based BQT, was approved only after 2021 and was therefore not well represented in the current study. Vonoprazan is more effective in suppressing acid production compared with PPIs [16], and data from RCTs have shown that vonoprazan‐based BQT is as effective as PPI‐based BQTs in eradicating *H. pylori* infections [25].

Analysis of pooled data from 24 RCTs indicated that the *H. pylori* eradication rate for BQTs was 81.3% [17], which is higher than that observed in the current study. This difference between RCTs and the real‐world study is likely due to greater patient adherence to the treatment regimen and monitoring in the controlled environment of an RCT, which cannot be replicated in a clinical setting. In fact, poor adherence has been associated with significantly lower eradication rates [26]. Moreover, in real‐world settings, it is possible that healthcare practices may not always adhere strictly to standardized guidelines, leading to differences in treatment outcomes. Variations in dosage and treatment duration used in RCTs compared with those administered in clinical practice may have contributed to differences in eradication rates. Analysis of data from the European Registry on *Helicobacter pylori* management (Hp‐EuReg) reveals that along with adherence, the treatment duration and prescribed PPI dose had a significant effect on *H. pylori* eradication [27]. Furthermore, in the current study, clarithromycin was used in a high proportion of patients receiving BQTs. Clarithromycin has been reported to have high resistance rates ranging from 20% to 50% in China [14].

The eradication rate of dual therapy was also lower at 57% compared with BQT in this study, although they are expected to be similar [28] and both regimens are recommended in China for initial and second eradication treatments [17]. This low eradication rate could be attributed to the deviations from guidelines in real‐world practice and the high resistance rate for certain frequently used antibiotics, such as levofloxacin [29], which was widely used by patients in this study and had an eradication rate of 60%.

Analyses of data from the Hp‐EuReg concurred with our study findings, showing an overall decrease in the prescription of triple therapies and an increase in the prescription of quadruple therapies from 2013 to 2023 in Europe [30]. While eradication rates across treatment types varied with indications, the effectiveness of PPI‐BQTs was > 90% for all indications for *H. pylori* investigations except gastroesophageal reflux disease. This was greater than the overall eradication rate of 72% observed in the current study and could be attributed to the difference in antibiotic resistance between China and European countries [29, 31].

It is proposed that for the eradication of *H. pylori* , a treatment regimen needs to have an eradication rate of 90%–95% to be considered as “good.” [32] While such a threshold may be considered as arbitrary and unrealistic in real‐world settings considering the high antibiotic resistance observed [33], findings from this study and others [19] suggest that current *H. pylori* eradication rates in China are far from satisfactory and lower than those observed globally. Factors that can influence the success of *H. pylori* eradication include gastric acid level, the eradication regimen chosen, duration of treatment, treatment adherence, and antibiotic resistance patterns in the region. We observed that among patients using amoxicillin and furazolidone–based BQT, the eradication rate was higher at 87% compared with that observed with amoxicillin and clarithromycin– and amoxicillin and levofloxacin–based quadruple therapies (76% and 78%, respectively). While few patients were prescribed amoxicillin and clarithromycin–based high‐dose dual therapies at the time of this study, the eradication rates for these were 80% and 44%, respectively. Thus, considering the increasing clarithromycin and levofloxacin resistance in the region, the choice of antibiotic combinations used for quadruple and dual therapies may be critical even with an empirical treatment approach.

Of concern, only 58% of enrolled patients with *H. pylori* infections received eradication therapy. Further, only 7% of patients had a record of efficacy evaluation testing. While this may be partly attributed to missing data in patient records, it is also likely that not all *H. pylori* infections are being treated, and among those that are treated, the eradication rate was not being assessed/confirmed. This is consistent with the responses from surveyed clinicians in China [15] and suggests the need for training and educational initiatives to ensure greater consensus and alignment with guidelines for managing *H. pylori* infections.

This study has a few limitations. First, only tertiary hospitals in urban areas in China were included; therefore, the results may not be completely generalizable to a rural population or patients treated in primary and secondary healthcare settings. Second, the inclusion of regions only in Eastern China may not capture the diversity of healthcare practices and patient characteristics in other parts of the country [8, 34]. Third, the study findings are limited to *H. pylori* diagnosis and treatment for the period from 2019 to 2021 and do not capture specific trends beyond this period. Limitations due to the descriptive, retrospective, and observational nature of the study should also be acknowledged. For instance, patients' medical records were only linked within a hospital because of privacy concerns. If a patient underwent diagnostic testing/efficacy evaluation testing outside of the targeted hospital, this would not be captured. The full medical history of patients was not available, and patient compliance to therapy was not known; however, compliance to eradication therapy is associated with *H. pylori* eradication rate [35].

## 5 Conclusions

In this real‐world retrospective observational study, patients with *H. pylori* infections were generally managed according to treatment guidelines. However, the presence of certain gaps between guideline recommendations and real‐world practices highlights the potential for improvement and standardization in clinical practice in the management of *H. pylori* infection such that patients receive the most effective and evidence‐based treatments to improve the eradication rate of *H. pylori* in China.

## Conflicts of Interest

Changqin Xu, Wensheng Pan, Yan Zhao, and Hongwei Xu declare no conflicts of interest. Pengfei Li, Fang Zhou, and Li Xie are employees of Takeda and hold Takeda stock options.

## Supporting information


**Table S1.** Patient disposition.
**Table S2.** Baseline demographic characteristics (ENR).
**Table S3.** Distribution of eradication therapy (and antibiotic combination used) by year of initially prescribed treatment (TAS).
**Figure S1.** Distribution of eradication therapy by the year when treatment was initially prescribed (TAS).
**Figure S2.** Diagnostic testing before eradication treatment by the year when treatment was initially prescribed (DAS).
**Figure S3.** Efficacy evaluation testing after eradication treatment by the year when treatment was initially prescribed (EEAS).
**Figure S4.** Duration from medication termination until efficacy evaluation testing by the year when treatment was initially prescribed (EEAS).
**Figure S5.** Eradication rate of *H. pylori* –positive patients who received eradication therapy, by the year when treatment was initially prescribed (EEAS).

## Data Availability

The data that support the findings of this study are available from the corresponding author upon reasonable request.
