# Supplementary material for: Diagnosis, Treatment Patterns and Eradication Success for Helicobacter pylori Infections in China: A Retrospective Observational Real‐World Study
Source: JGH Open. 2025 Oct 10;9(10):e70232. doi: 10.1002/jgh3.70232 (PMC12514013; doi:10.1002/jgh3.70232)
Supplement: Supplementary file 1 — Table S1. Patient disposition. Table S2. Baseline demographic characteristics (ENR). Table S3. Distribution of eradication therapy (and antibiotic combination used) by year of initially prescribed treatment (TAS). Figure S1. Distribution of eradication therapy by the year when treatment was initially prescribed (TAS). Figure S2. Diagnostic testing before eradication treatment by the year when treatment was initially prescribed (DAS). Figure S3. Efficacy evaluation testing after eradication treatment by the year when treatment was initially prescribed (EEAS). Figure S4. Duration from medication termination until efficacy evaluation testing by the year when treatment was initially prescribed (EEAS). Figure S5. Eradication rate of H. pylori –positive patients who received eradication therapy, by the year when treatment was initially prescribed (EEAS). [file JGH3-9-e70232-s001.docx]

**Supplemental Material**

**Methods**

**Ethics statement**

The study was conducted in accordance with the protocol and executed in line with all applicable regulations and guidelines, including the Declaration of Helsinki, Chinese good clinical practices, and any local regulations and guidelines of China.

Informed consent was not required for this study as EMR data were collected retrospectively. A waiver regarding participant consent was applied from each participating hospital, and this decision was stated in the Ethical Approval Letter obtained from each participating hospital.

**Results**

**Supplemental Table 1**. Patient disposition

|  | **Total  (N=22,971)** |
| --- | --- |
|  | **n (%)** |
| All patients enrolled set (ENR)* | 22,971 (100.0) |
| Diagnostic analysis set (DAS) | 15,712 (73.8) |
| Treatment analysis set (TAS) | 13,233 (57.6) |
| Efficacy evaluation analysis set (EEAS) | 1,626 (7.1) |

*The extracted raw data consisted of 23,000 patients. One patient was excluded because the index date fell outside the identification period. Three patients in Shandong and 25 patients in Zhejiang were excluded from the study because they were <18 years of age at the index date, which did not meet the inclusion criteria.

Index date: Date of the first diagnosis or the first date of a positive result of *H. pylori* diagnostic testing, whichever came first during the patient identification period (January 01, 2019, to December 31, 2021).

**Supplemental Table 2**. Baseline demographic characteristics (ENR)

|  | **Total  (N=22,971)** |
| --- | --- |
| **Characteristics** | **n** (%) |
| **Age at index (years)** |  |
| Mean (SD) | 44.2 (14.2) |
| Median | 42 |
| Q1, Q3 | 32, 56 |
| Min, max | 18, 97 |
| Missing, n | 0 |
| **Age categories (years)** |  |
| Nx | 22,971 (100.0) |
| ≥18 and ≤44 | 12,376 (53.9) |
| ≥45 and ≤59 | 6,680 (29.1) |
| ≥60 | 3,915 (17.0) |
| Missing, n | 0 (0.0) |
| **Sex** |  |
| Nx | 22,951 (99.9) |
| Male | 10,416 (45.4) |
| Female | 12,535 (54.6) |
| Missing, n | 20 (0.1) |
| **Health insurance** |  |
| Nx | 22,720 (98.9) |
| With medical insurance | 14,571 (64.1) |
| Full self-pay | 7,322 (32.2) |
| Others* | 827 (3.6) |
| Missing, n | 251 (1.1) |
| **Geographic area** |  |
| Nx | 22,971 (100.0) |
| Shanghai city | 8,000 (34.8) |
| Shandong province | 6,996 (30.5) |
| Zhejiang province | 7,975 (34.7) |
| Missing, n | 0 (0.0) |

ENR, all patients enrolled set; max, maximum; min, minimum; Nx, number of patients without missing values; Q, quarter; SD, standard deviation.

The denominator of the percentage calculation does not include the missing value.

*“Others” includes municipal administration and communication all-purpose card, other social medical insurance, publicly funded medical care, interprovincial outpatient insurance, interprovincial (residents) insurance, interprovincial in allopatry insurance, intraprovincial in allopatry insurance, intraprovincial chronic disease insurance, interprovincial (employees) insurance, and designated entities insurance.

**Supplemental Table 3**. Distribution of eradication therapy (and antibiotic combination used) by year of initially prescribed treatment (TAS)

|  | **Year 2019 (N=4,492)** | **Year 2020 (N=4,125)** | **Year 2021 (N=4,616)** |
| --- | --- | --- | --- |
|  | **n** (%)* | **n** (%)* | **n** (%)* |
| **Total usage count of all standard eradication therapies** | 4,492 | 4,125 | 4,617^†^ |
| **Bismuth quadruple therapy** | 4,368 (97.2) | 3,299 (80.0) | 3,634 (78.7) |
| Amoxicillin and clarithromycin–based therapy | 1,800 (41.2) | 1,324 (40.1) | 1,252 (34.5) |
| Amoxicillin and levofloxacin–based therapy | 1,021 (23.4) | 516 (15.6) | 470 (12.9) |
| Amoxicillin and furazolidone–based therapy | 689 (15.8) | 480 (14.5) | 905 (24.9) |
| Clarithromycin and levofloxacin–based therapy | 515 (11.8) | 576 (17.5) | 246 (6.8) |
| Levofloxacin and metronidazole–based therapy | 141 (3.2) | 76 (2.3) | 183 (5.0) |
| Clarithromycin and furazolidone–based therapy | 109 (2.5) | 163 (4.9) | 100 (2.8) |
| Other bismuth quadruple therapy | 93 (2.1) | 164 (5.0) | 478 (13.2) |
| **Triple therapy** | 105 (2.3) | 551 (13.4) | 343 (7.4) |
| Amoxicillin and clarithromycin–based therapy | 43 (41.0) | 18 (3.3) | 22 (6.4) |
| Clarithromycin and levofloxacin–based therapy | 30 (28.6) | 462 (83.9) | 110 (32.1) |
| Amoxicillin and levofloxacin–based therapy | 22 (21.0) | 7 (1.3) | 24 (7.0) |
| Other triple therapy | 10 (9.5) | 64 (11.6) | 187 (54.5) |
| **Dual therapy** | 19 (0.4) | 275 (6.7) | 640 (13.9) |
| Levofloxacin-based therapy | 11(57.9) | 216 (78.5) | 192 (30.0) |
| Clarithromycin-based therapy | 3 (15.8) | 45 (16.4) | 29 (4.5) |
| Amoxicillin-based therapy | 2 (10.5) | 8 (2.9) | 15 (2.3) |
| Ornidazole-based therapy | 1 (5.3) | 4 (1.5) | 393 (61.4) |
| Other dual therapy | 2 (10.5) | 2 (0.7) | 11 (1.7) |

TAS, treatment analysis set.

*The percentage is based on either the total usage count of all standard eradication therapies taken by eligible patients for the overall therapy results (shaded rows) or on the total usage count of bismuth quadruple/triple/dual therapy for individual antibiotic combination results for each year.

^†^One patient changed the treatment pattern from triple therapy (ilaprazole, clarithromycin, levofloxacin) to dual therapy (ilaprazole, ornidazole) after recurrence.

**Supplemental Figure 1**. Distribution of eradication therapy by the year when treatment was initially prescribed (TAS)


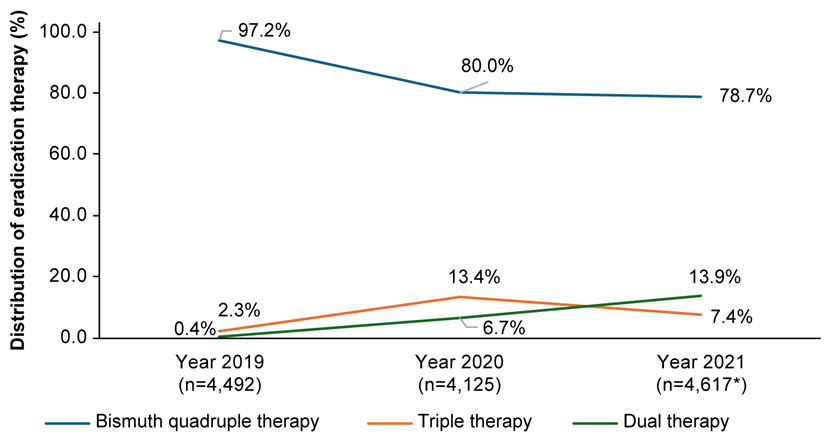


TAS, treatment analysis set.

*One patient changed the treatment pattern from triple therapy (ilaprazole, clarithromycin, levofloxacin) to dual therapy (ilaprazole, ornidazole) after recurrence.

**Supplemental Figure 2**. Diagnostic testing before eradication treatment by the year when treatment was initially prescribed (DAS)

**
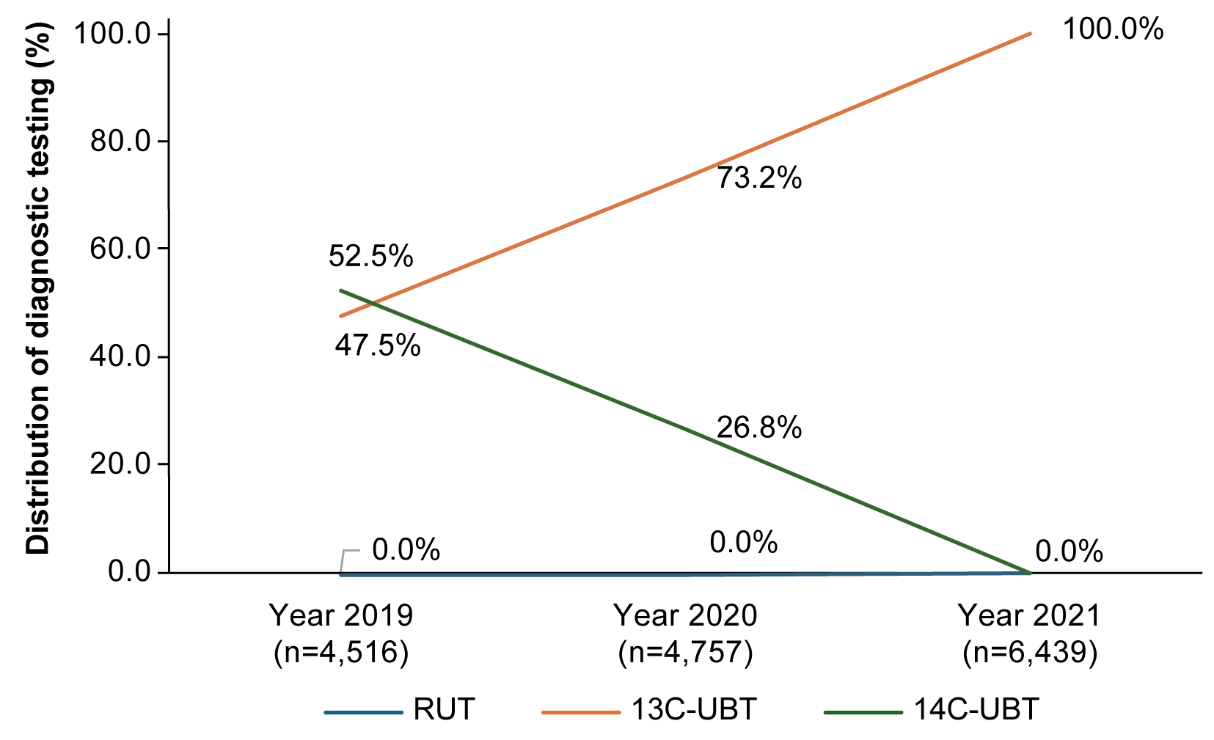
**

DAS, diagnosis analysis set; RUT, rapid urease test; UBT, urea breath test.

One patient had both RUT and UBT data at the index date.

**Supplemental Figure 3**. Efficacy evaluation testing after eradication treatment by the year when treatment was initially prescribed (EEAS)


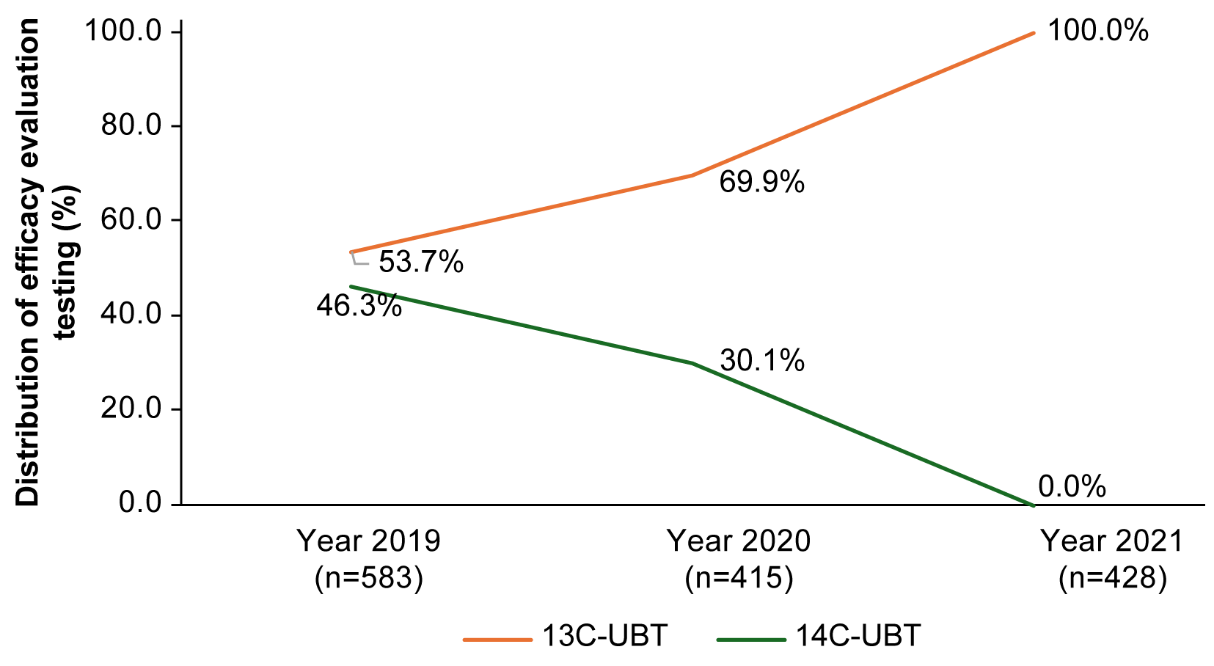


EEAS, efficacy evaluation analysis set; UBT, urea breath test.

**Supplemental Figure 4**. Duration from medication termination until efficacy evaluation testing by the year when treatment was initially prescribed (EEAS)


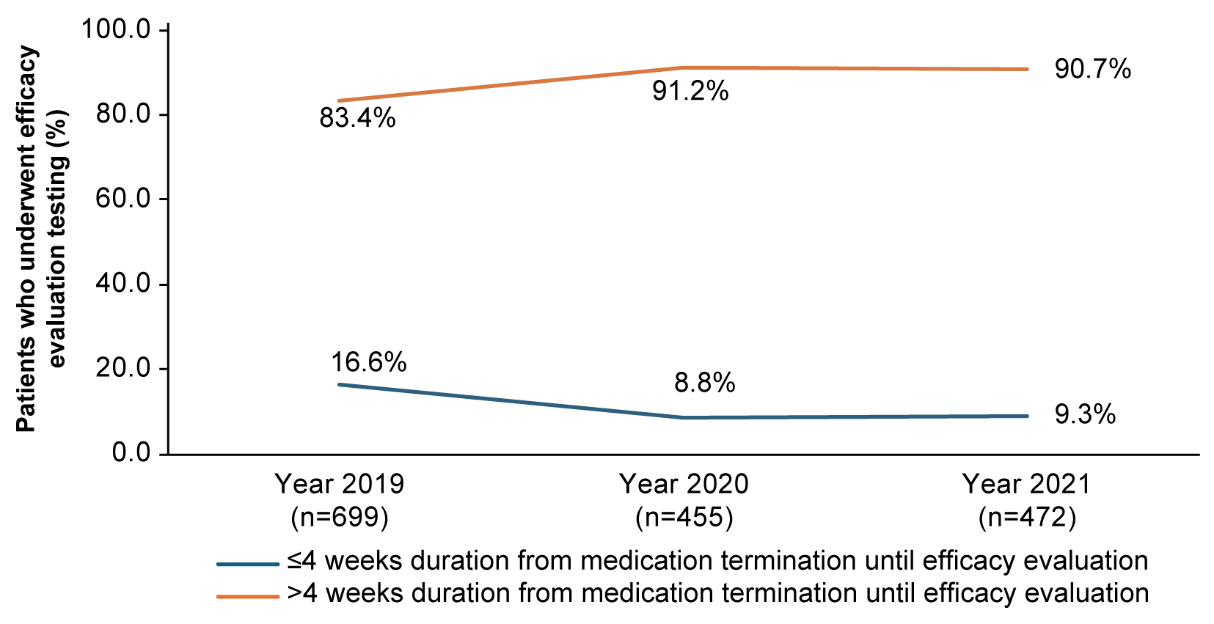


EEAS, efficacy evaluation analysis set.

**Supplemental Figure 5**. Eradication rate of *H. pylori*–positive patients who received eradication therapy, by the year when treatment was initially prescribed (EEAS)


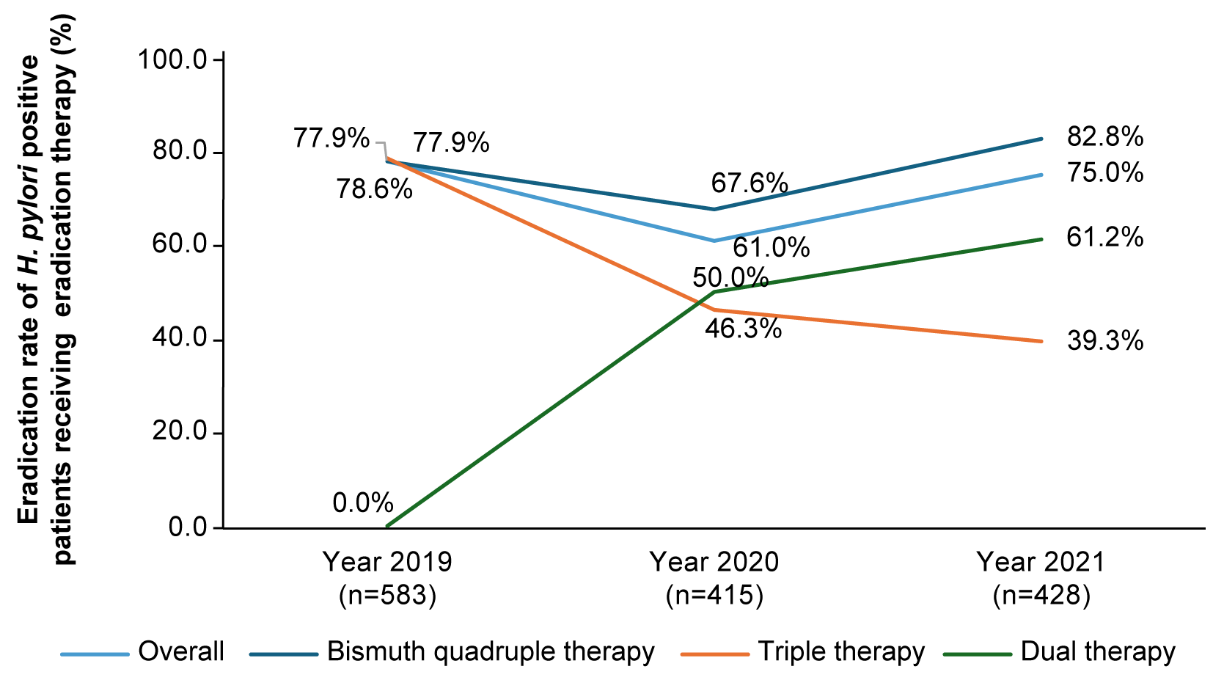


EEAS, efficacy evaluation analysis set.
